# Effect of immunization during pregnancy and pre-existing immunity on diphtheria-tetanus-acellular pertussis vaccine responses in infants

**DOI:** 10.1080/22221751.2023.2204146

**Published:** 2023-05-03

**Authors:** Aapo Knuutila, Alex-Mikael Barkoff, Lauri Ivaska, Elina Tenhu, Johanna Teräsjärvi, Pieter van Gageldonk, Annemarie Buisman, Jussi Mertsola, Qiushui He, PERISCOPE consortium

**Affiliations:** a Research Center for Cancer, Infections and Immunity, Institute of Biomedicine, Centre for Infections and Immunity, University of Turku, Turku, Finland; b Department of Paediatric and Adolescent Medicine, Turku University Hospital, and University of Turku, Turku, Finland; c InFLAMES Research Flagship Center, University of Turku, Turku, Finland; d National Institute for Public Health and the Environment (RIVM), Centre for Infectious Disease Control, Bilthoven, the Netherlands

**Keywords:** Immunization in pregnancy, primary vaccination, pertussis, antibodies, pertussis toxin neutralization, memory B cells, infants

## Abstract

Immunization during pregnancy (IP) against pertussis is recommended in many countries to protect infants. Although maternal antibodies can influence the infants’ antibody responses to primary vaccinations, their effect on the development of functional antibodies and B cells remain poorly studied. We investigated the maternal immune response to IP and the effect of IP and pre-existing antibodies on infants’ primary vaccine responses in an open-label, non-randomized trial. Forty-seven mothers received tetanus-diphtheria-acellular pertussis (Tdap) vaccine during pregnancy, and 22 mothers were included as controls. Sixty-nine infants received primary doses of DTaP at three and five months of age. Geometric mean concentrations of antibodies to pertussis toxin, filamentous haemagglutinin, pertactin, diphtheria, and tetanus toxins, pertussis toxin neutralizing antibodies (PTNAs), and plasma and memory B-cell frequencies were studied at delivery, and at three, five and six months. Levels of antibodies, PTNAs, and frequencies of memory B-cells were significantly increased at delivery and up to six months after in mothers with IP compared to those without IP (all *p* < 0.05, except for PT-specific memory B-cells). In vaccinated pregnant women, high pre-existing antibody levels were positively correlated with higher antibody responses after IP. IP blunted the infants’ antibody and plasma B-cell responses to all vaccine antigens, except for tetanus toxin. This blunting effect was the strongest in infants with high concentrations of maternal antibodies. In conclusion, IP resulted in significantly higher concentrations of antibodies in infants up to three months of age (all *p* < 0.05); but was associated with blunting of various infants’ vaccine responses.

## Introduction

Pertussis is a highly contagious respiratory disease caused by the bacterium *Bordetella pertussis*. The disease is potentially life-threatening especially to unvaccinated infants. Nationwide pertussis epidemics with infant deaths during 2008–2014 in Australia, the US, and the UK led these countries to introduce immunization in pregnancy (IP) against pertussis to protect vulnerable infants [1–4]. Studies have shown that IP is safe both for the mothers and for their children, and an effective tool to protect infants before their first dose of pertussis primary vaccination [2,4–6].

Studies have shown that IP decreases the antibody responses of infants to their primary diphtheria-tetanus-acellular pertussis (DTaP) vaccination. This phenomenon is called blunting. Blunting of vaccine responses has been observed in IgG-antibody concentrations to different pertussis vaccine antigens, specifically, pertussis toxin (PT), filamentous haemagglutinin (FHA), and pertactin (PRN), and diphtheria toxin (DT) [5,7]. The extent of blunting seems to depend on which vaccines are used for the infants [8]. It has been shown that maternally transferred antibodies blunt the quantity but not the quality of PT antibodies in children induced by their primary vaccination series [9,10]. To our knowledge, no studies regarding PT-neutralizing antibodies (PTNAs) have been conducted among infants whose mothers received IP. In addition, there are only a few studies that have focused on the effect that IP has on the developing cell-mediated immunity in infants. These studies have demonstrated decreased cytokine responses in infants born to vaccinated mothers [11,12]. Although many studies have shown that maternal antibodies blunt the infants’ antibody responses to pertussis primary vaccinations, little is known about their impact on the infants’ PTNAs and B cells.

In this study, we investigated the effect of the existing immunity, such as antibodies or B-cells, on the development of vaccine-induced immunity after pertussis vaccination both in pregnant women and infants. This study was conducted as part of the PERISCOPE project, which aims for better control of pertussis [13].

## Materials and methods

### Study design, participants, and study procedures

We conducted a prospective, interventional, open-label controlled study to evaluate the effect of tetanus-diphtheria-acellular pertussis, containing PT, FHA, and PRN, (Tdap) (Boostrix, GSK) vaccination during pregnancy at 30–35 weeks of pregnancy on DTaP vaccine responses of the infants. The primary outcome was the PT-specific antibody geometric mean concentration (GMC) in infants born to vaccinated mothers (Arm 1) in comparison with infants born to mothers not vaccinated during pregnancy (Arm 2) at six months of age. Other outcomes in the infants included antibody concentrations to FHA, PRN, DT, and tetanus toxin (TT), as well as PTNAs, and memory and plasma B-cell levels at delivery and at three, five and six months of age. The trial was registered in the EU Clinical Trial database (EudraCT number 2019-001986-34) and was approved by the Ethics Committee of the Hospital District of Southwest Finland (ETMK Dnro 67 /1800/2019). Written informed consent was obtained from all participants.

Exclusion criteria for mothers were age < 18 years, a history of systemic disease, Tdap vaccination during the past two years, immunosuppressive treatment during pregnancy, and HIV positivity. Pregnant mothers were recruited from maternity clinics in the Turku area from January 2020 to March 2021. After providing written informed consent, pregnant women could choose if they wanted to participate in the study in Arm 1 or Arm 2. Exclusion criteria for infants were birth before 37 weeks of gestation, birth weight < 2,500 g, safeguarding of the infant in place, participation in other vaccine or drug trials, major congenital defects, serious systemic disease, immunodeficiency, receipt of immune-suppressants or immune modifying drugs, receipt of immunoglobulin or blood products, history of allergy to any component of the vaccine, history of pertussis, and lack of immunization with study vaccine.

Apart from the Tdap booster vaccination during pregnancy, women and infants in both arms of the trial underwent the same study procedures. Blood samples from mothers were collected before maternal vaccination, at 48 h postpartum, and six months postpartum. In addition, cord blood samples were collected. Study infants received their vaccines at the study clinic according to the national immunization schedule in Finland: oral rotavirus vaccine (RotaTeq, MSD) at two, three, and five months of age; and 10-valent pneumococcal conjugate vaccine (Synflorix, GSK) at three and five months of age. Instead of the pentavalent DTaP-IPV-Hib vaccine included in the Finnish national programme, study infants received hexavalent DTaP-IPV-Hib-HepB vaccine (Infanrix hexa, GSK) at three and five months of age to give better comparisons to other PERISCOPE studies [13]. Blood samples from infants were collected before the first pertussis vaccine at three months, seven days after the five-month vaccine, and at six months of age.

### Antibody analyses

Serum IgG concentrations against purified PT, FHA, PRN, Fimbriae (FIM) 2/3, DT, and TT were quantified in independent duplicates using a fluorescent-bead-based multiplex immunoassay as previously described [14,15]. In brief, purified antigens conjugated with fluorescent microbeads were incubated with serum samples in two dilutions (1/200 and 1/4,000) and in-house serum reference in a dilution series. The measurement of the IgG antibody concentrations was performed with a Luminex Flexmap 3D in combination with xPONENT 4.3 (DiaSorin/Luminex), and mean fluorescence intensity was converted to IU/ml by interpolation from a five-parameter logistic standard curve using BioPlex Manager 6.2 (Bio-Rad Laboratories). Antibody concentrations below the limit of detection were assigned the lower limit of detection value for that antigen.

PTNAs were determined by a CHO cell assay as described previously [16,17]. Briefly, 5,000 or 10,000 CHO cells were first mixed with 0.84 ng/ml of purified native PT and incubated for 3–4 h. Serum was added to the wells in a dilution series (1:8–1:4,096; later interpreted as 8–4,096 titres), and the plate was incubated for up to 30 h. The wells were evaluated visually after 24 h with Incucyte or Incucyte ZOOM instruments (Essen Bioscience, Ann Arbor, MI, USA). The neutralizing titre was reported as the reciprocal of the corresponding serum dilution in the last well without clustering of CHO-cells, and samples without neutralization were assigned a value of “4”.

### B-cell analyses

Peripheral blood mononuclear cells (PBMCs) were isolated from whole blood and tested for quantification of memory B-cells by antigen-specific ELISpot as described previously [18]. The frozen PBMCs were thawed and then pre-stimulated in culture media including IL-2, IL-10, and CpG for five days, after which the cells were harvested. The ELISpot plates were coated with native PT, FHA, and PRN antigens. Additionally, FIM- and TT-specific memory B-cells were tested from the mothers. PBS was used as a negative control (blank) and total anti-human IgG as a positive control. A total of 200,000 pre-stimulated PBMCs per well were used and tested in duplicate. Similar to memory B-cells, frequencies of infants’ plasma B-cells were measured one week after the second vaccine dose; however, fresh PBMCs were stimulated directly with the vaccine antigens, excluding the five-day pre-stimulus.

ELISpot plates were visualized with goat anti-human IgG (Merck-Millipore, 401442) and SIGMAFAST BCIP/NBT alkaline phosphatase substrate (Sigma-Aldrich, B5655) and measured with Bioreader 3000 ELISpot Reader (BIO-SYS GmbH, Karben, Germany). Geometric mean frequencies were calculated for each antigen of B-cells per 100,000 PBMCs and the background frequency from the negative control was subtracted per individual. The lowest number of detectable spots was 0.25, and lower numbers were set at a value of 0.1 for further analysis. The median spot frequency in the blank wells was 0 per 100,000 PBMCs.

### Statistics

The sample size calculation for the study was based on earlier results [5]. With 40 infants in both study arms, the statistical power to show a difference of at least 47 units in the PT-IgG GMCs between the groups at six months of age is 99%. The power calculations were based on the probability of a 95% confidence interval of the difference of GMCs not covering a value of “0” under an alternative hypothesis based on a previously reported difference of 47 units in GMC, corresponding to a 1 SD (0.37) difference on the log-scale (base 10), in children at six months of age. Due to COVID restrictions, study recruitment did not proceed as expected. Therefore, we decided to settle for lower recruitment aims and not prolong the recruitment period substantially.

To test for differences between Arm 1 and Arm 2, the Mann–Whitney *U* test was used. Three-month time point was used as the baseline for evaluating infants’ vaccine responses. To evaluate the effect of the mother’s existing memory of vaccination antigens on maternal and infant antibody levels, an analysis by grouping individuals based on high and low baseline antibodies was performed. The Wilcoxon signed-rank test was used to assess differences in antibody concentrations between the study time points. The correlation between the study parameters was evaluated using Pearson’s or Spearman’s R when applicable. The analyses were performed using IBM SPSS Statistics for Windows version 28.0 (IBM Corp., Armonk, NY, USA). Statistical significance was set at *p* < 0.05.

## Results

**Figure 1.**
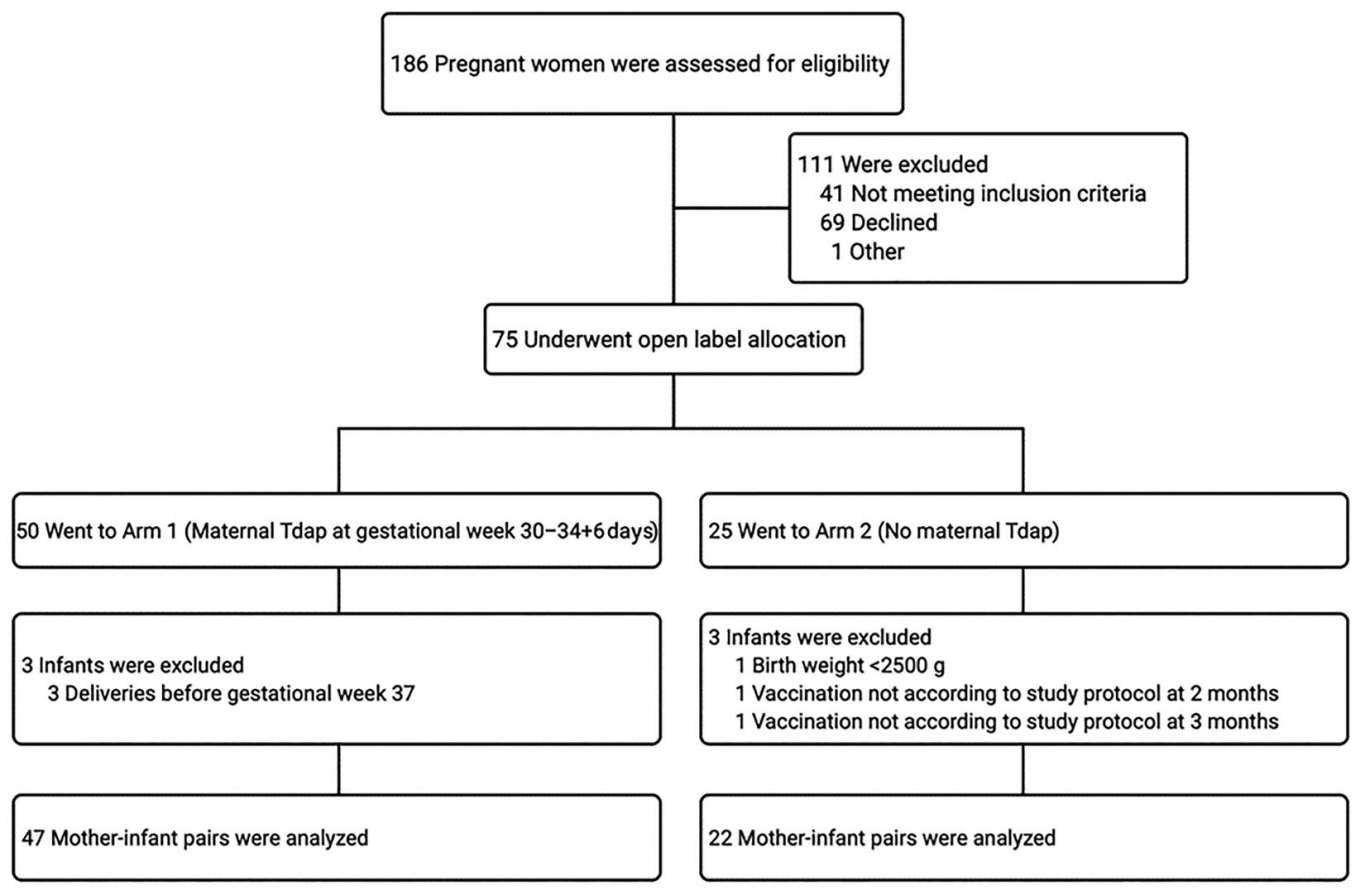
Study flow chart. Abbreviations: Tdap, tetanus-diphtheria-acellular pertussis vaccine.

After screening 186 pregnant women, 75 underwent the open-label allocation in one of the study arms. In total, 69 mother-infant pairs completed the study, of which 47 mothers and infants were included in Arm 1 and 22 mothers and infants in Arm 2 (Figure 1). The baseline characteristics of the study participants are shown in Table 1.

**Table 1.** Baseline characteristics of study participants (*n* = 69 mother-infant pairs).

	Arm 1, Tdap during pregnancy (*n* = 47)	Arm 2, No Tdap during pregnancy (*n* = 22)
**Women**		
Median age at delivery, *yr* [IQR]	31.4 [29.1–34.8]	31.9 [28.2–34.6]
Gestational age at delivery, *wk* [IQR]	40.1 [39.0–41.0]	40.2 [39.4–41.1]
Gestational age at vaccination or inclusion to the study, *wk* [IQR]	31.0 [30.4–32.3]	30.9 [30.3–31.9]
Median time between IP or inclusion to the study and delivery, *wk* [IQR]	8.4 [7.6–9.6]	9.4 [7.6–10.5]
Caesarean section, *n* (%)	6 (13)	3 (14)
**Infants**		
Median birth weight, *g* [IQR]	3,684 [3,270–3,930]	3,605 [3,203–3,979]
Sex (male), *n* (%)	22 (47)	14 (64)
Admitted to neonatal intensive care unit, *n* (%)	1 (2)	2 (9)
Started breastfeeding after delivery, *n* (%)	47 (100)	21 (96)
Median duration of breastfeeding, *wk* [IQR]	17.4 [9.1–19.6]	17.4 [7.7–17.9]

Tdap, tetanus-diphtheria-acellular pertussis vaccine; IQR, interquartile range; IP, immunization during pregnancy.

### Safety of the study vaccines

Vaccination in pregnant women was well tolerated. Altogether, 50 women received the Tdap vaccine of whom three were later excluded from the study cohort based on the predefined exclusion criteria (preterm delivery < 37 weeks). No serious adverse events related to study interventions or sudden unexpected serious adverse reactions were identified in any study participant.

### Igg antibody concentrations and PTNAs

#### Mothers

The baseline IgG GMCs were low in both study arms, although GMCs to PT, FHA, and PRN were slightly higher in Arm 2 (Table 2). A small proportion of mothers, 4/47 (8.5%) of participants in Arm 1 and 3/22 (13.6%) in Arm 2, had anti-PT IgG above 50 IU/ml, suggestive of a previous pertussis infection within a few years [19,20]. After the Tdap booster vaccination, the vaccine antigen-specific IgG GMCs were significantly increased against all vaccine antigens in Arm 1 (*p* < 0.001). However, 12/47 (26%) of mothers in Arm 1 had a low increase in anti-PT IgG (< 25 IU/ml increase), and one mother showed no response to PRN, TT, and DT. Similarly, 11%, 13%, 15%, and 6% mothers had a low increase of antibodies to FHA, PRN, DT, and TT (less than 50, 50, 0.125, and 0.5 IU/ml), respectively. Mothers in Arm 1 had a minor decrease in their GMCs from delivery up to the six-month time point, whereas mothers in Arm 2 had an increase in their GMCs. There was no increase in anti-FIM IgG antibody concentration in either of the study arms.

**Table 2.** Geometric mean values of mothers’ IgG antibodies, pertussis toxin neutralizing antibodies (PTNA), and memory B-cell frequencies before and after Tdap vaccination. Significantly higher levels of anti-PT, FHA, PRN, DT, TT, and PTNA titres as well as higher frequencies of memory B cells (aside from PT at 6 months) to PT, FHA, PRN, and TT were found at delivery and 6 months in Arm 1 than Arm 2 (all *p* values < 0.05).

	No. Subjects	IgG antibodies IU/ml (95% CI)						PTNA titre	B-cell ELISPOTs per 100,000 cells (95% CI)				
Time point	(IgG, PTNA, ELISPOT)	PT	FHA	PRN	FIM	DT	TT	(95% CI)	PT	FHA	PRN	FIM	TT
Pregnancy													
Arm 1	47, 47, 47	5.5 (3.4–9.0)	17.3 (11.7–25.6)	15.3 (10.1–23.3)	7.4 (4.6–11.9)	0.1 (0.1–0.2)	1.4 (1.2–1.8)	32.5 (20.5–51.3)	0.3 (0.2–0.5)	0.3 (0.2–0.4)	0.5 (0.3–1.0)	ND**	0.6 (0.4–1.0)
Arm 2	22, 22, 22*	11.9 (7.2–19.7)	27.0 (15.3–47.6)	41.6 (25.1–68.9)	7.3 (4.0–13.4)	0.1 (0.1–0.2	1.5 (1.0–2.2)	28.2 (16.8–47.3)	0.3 (0.2–0.6)	0.3 (0.1–0.5)	0.8 (0.3–1.9)	ND	0.7 (0.2–3.1)
Delivery													
Arm 1	47, 46, 47	54.9 (36.0–84.0)	201.1 (149.9–269.8)	251.4 (177.8–355.6)	6.4 (3.9–10.4)	0.7 (0.6–0.9)	4.9 (4.0–6.0)	140.1 (93.3–210.3)	1.0 (0.6–1.6)	2.0 (1.2–3.3)	3.7 (2.3–5.7)	ND	1.5 (0.8–2.5)
Arm 2	22, 22, 22*	10.2 (6.1–16.9)	20.4 (11.6–35.7)	27.5 (15.0–50.4)	6.5 (3.5–12.0)	0.1 (0.1–0.2)	1.2 (0.8–1.7)	37.5 (23.8–59.0)	ND	ND	ND	0.3 (0.1–0.8)	0.6 (0.2–2.8)
6 months													
Arm 1	47, 47, 47	48.1 (31.6–73.3)	182.4 (139.5–238.5)	247.5 (168.4–363.9)	11.3 (6.8–18.7)	0.6 (0.5–0.8)	4.4 (3.7–5.2)	169.4 (116.8–245.6)	0.5 (0.3–0.9)	0.9 (0.6–1.5)	2.0 (1.2–3.3)	0.3 (0.2–0.4)	1.3 (0.8–2.3)
Arm 2	22, 22, 22*	18.3 (10.7–31.2)	32.5 (17.8–59.4)	50.2 (26.8–94.0)	10.0 (5.2–19.0)	0.2 (0.1–0.4)	2.2 (1.4–3.3)	42.5 (24.7–73.1)	0.3 (0.1–0.5)	ND	0.4 (0.2–0.9)	0.3 (0.1–0.5)	0.7 (0.1–3.2)

*Only 10 samples were tested for FIM and TT ELISPOT

**Not defined, GM below the lower limit of detection

PTNA titres increased significantly in Arm 1 (*p* < 0.001) and remained significantly higher up to the six-month time point (*p* < 0.001) (Table 2). In Arm 2, PTNA values did not change throughout the study. PTNAs were well correlated with anti-PT IgG (Spearman R = 0.723).

#### Infants

Overall, IgG GMCs between the infants and mothers were well correlated at birth (Pearson R > 0.838 for all individual vaccine antigens). The GMCs were significantly higher in Arm 1 than in Arm 2 (Table 3) in cord blood to all vaccine antigens (*p* < 0.001). Antibodies remained significantly higher in Arm 1 in comparison to Arm 2 for up to three months, despite a strong decrease in GMCs in Arm 1 from delivery (*p* < 0.001). After the first two primary doses, an increase in PT antibodies was noted in both arms from three to six months. However, the increases in GMCs were significant only in Arm 2 (*p* < 0.001). At six months, infants in Arm 2 had significantly higher GMCs to all vaccine antigens (*p* < 0.05) except TT (*p *= 0.709) in comparison to Arm 1. On average, 28.6% of infants in Arm 1 had higher than a two-fold increase of antibodies to a vaccine antigen at six months, whereas the portion was 76.0% in Arm 2.

**Table 3.** Geometric mean values of infants’ IgG antibodies, pertussis toxin neutralizing antibodies (PTNA), and pertussis specific memory (delivery, three months, six months) and plasma (five months + seven days) B–cell frequencies before and after DTaP vaccination.

	No. Subjects	IgG antibodies IU/ml (95% CI)						PTNA titre	B–cell ELISPOTs per 100,000 cells (95% CI)		
Time point	(IgG, PTNA, ELISPOT)	PT	FHA	PRN	FIM	DT	TT	(95% CI)	PT	FHA	PRN
Cord blood											
Arm 1	46, 44, 43	93.7 (60.9–144.3)	347.8 (258.8–467.3)	375.7 (267.1–528.6)	9.4 (5.6–15.8)	1.1 (0.9–1.4)	8.7 (7.1–10.6)	178.2 (125.9–252.1)	ND**	ND	ND
Arm 2	22, 21, 19	18.5 (10.9–31.2)	40.1 (22.4–71.7)	48.6 (25.2–93.5)	11.2 (5.7–22.2)	0.2 (0.1–0.3)	2.3 (1.5–3.5)	40.3 (23.3–69.8)	ND	ND	ND
3 months											
Arm 1	44, 45, 44	15.1 (9.7–23.6)	65.3 (46.4–86.2)	69.1 (49.4–96.6)	1.8 (1.1–3.1)	0.2 (0.1–0.2)	1.4 (1.2–1.8)	65.0 (48.0–88.0)	ND	ND	ND
Arm 2	22, 22, 19	3.6 (2.1–6.1)	6.9 (3.8–12.4)	8.5 (4.3–16.8)	1.8 (0.8–3.9)	0.0 (0.0–0.1)	0.5 (0.3–0.8)	28.2 (18.3–43.4)	ND	ND	ND
5 months + 7 days											
Arm 1	47, –, 38*	45.2 (36.7–55.6)	43.8 (37.7–51.0)	40.1 (31.8–50.7)	0.7 (0.4–1.2)	0.1 (0.1–0.2)	1.1 (1.0–1.3)	–	0.3 (0.2–0.5)	0.3 (0.2–0.5)	ND
Arm 2	18, –, 18	100.3 (60.5–166.1)	64.6 (44.2–94.4)	60.2 (31.3–115.8)	0.6 (0.3–1.2)	0.5 (0.3–0.8)	1.0 (0.7–1.6)	–	0.6 (0.3–1.2)	0.8 (0.4–1.8)	0.6 (0.3–1.3)
6 months											
Arm 1	46, 46, 41	68.2 (55.8–83.3)	57.5 (45.7–72.5)	31.7 (24.9–40.3)	0.5 (0.3–0.8)	0.1 (0.1–0.2)	1.3 (1.1–1.6)	113.5 (90.1–142.9)	ND	0.4 (0.3–0.6)	ND
Arm 2	22, 22, 21	141.9 (98.9–203.6)	97.4 (67.8–139.9)	54.3 (32.1–91.9)	0.4 (0.2–0.8)	0.5 (0.3–0.8)	1.4 (1.0–2.0)	116.5 (80.4–168.8)	ND	0.9 (0.4–1.9)	ND

*37 for PRN

**Not defined, GM below the lower limit of detection

Similar to anti-PT IgG, PTNAs were significantly higher at delivery and three months later in Arm 1 (Table 3). Unlike anti-PT IgG, PTNA titres did not differ between the study arms at six months. However, the fold increase of PTNA GM values from three to six months was 4.13 in Arm 2 and 1.75 in Arm 1 (*p *< 0.001).

### Memory and plasma B-cells

#### Mothers

In comparison to Arm 2, mothers in Arm 1 had significantly increased numbers of memory B-cells to all vaccine antigens at delivery (*p* < 0.05). The increased frequencies remained significantly higher for all vaccine antigens except for PT at six months. The frequency of PT-specific memory B-cells in Arm 1 was higher at the six-month time point compared to baseline, but did not reach statistical significance (*p *= 0.098) (Table 3).

At delivery, 26 (55%), 35 (74%), 34 (72%), and 24 (51%) mothers in Arm 1 had higher numbers of memory B-cell than before vaccination to PT, PRN, FHA, and TT, respectively. At the six-month time point, 16 (34%), 26 (55%), 27, and 27 (57%) mothers had more PT, PRN, FHA, and TT-specific memory B-cells, respectively, in comparison to the baseline.

#### Infants

The number of memory B-cells of infants was low at all time points with all study antigens (Table 3). Significantly higher GM plasma B-cell numbers were noted in Arm 2 for FHA (*p* = 0.02) compared to Arm 1. A similar trend was also noted for PRN (*p* = 0.06). A nearly two-fold higher frequency of PT plasma B-cells was found in Arm 2 compared to Arm 1, although the difference was not statistically significant. Notably, the proportion of infants with at least a frequency of five plasma B-cells varied between antigens from 13.5%, 0.0%, and 13.5% for PT, PRN, and FHA in Arm 1, and to 33.3%, 33.3%, and 39.0% in Arm 2, respectively.

At six months, significantly higher GM memory B-cell frequencies were observed in Arm 2 for FHA (*p* = 0.044) (Table 3), whereas there were no notable memory B-cells detected specific for PT and PRN in either study arm. Memory B-cell frequencies were significantly elevated only for FHA in Arm 2 at six months compared to baseline (*p* < 0.001 Wilcoxon signed-rank test). At six months, only 4.9%, 2.4%, and 22.0% of infants had at least a frequency of five specific memory B-cells for PT, PRN, and FHA, respectively, in Arm 1, whereas the respective proportions were 0.0%, 4.8%, and 42.9% in Arm 2. No correlations were observed between the frequencies of plasma B-cells and memory B-cells and the three antigens.

### Effect of pre-existing antibodies on mothers’ vaccination responses

**Figure 2.**
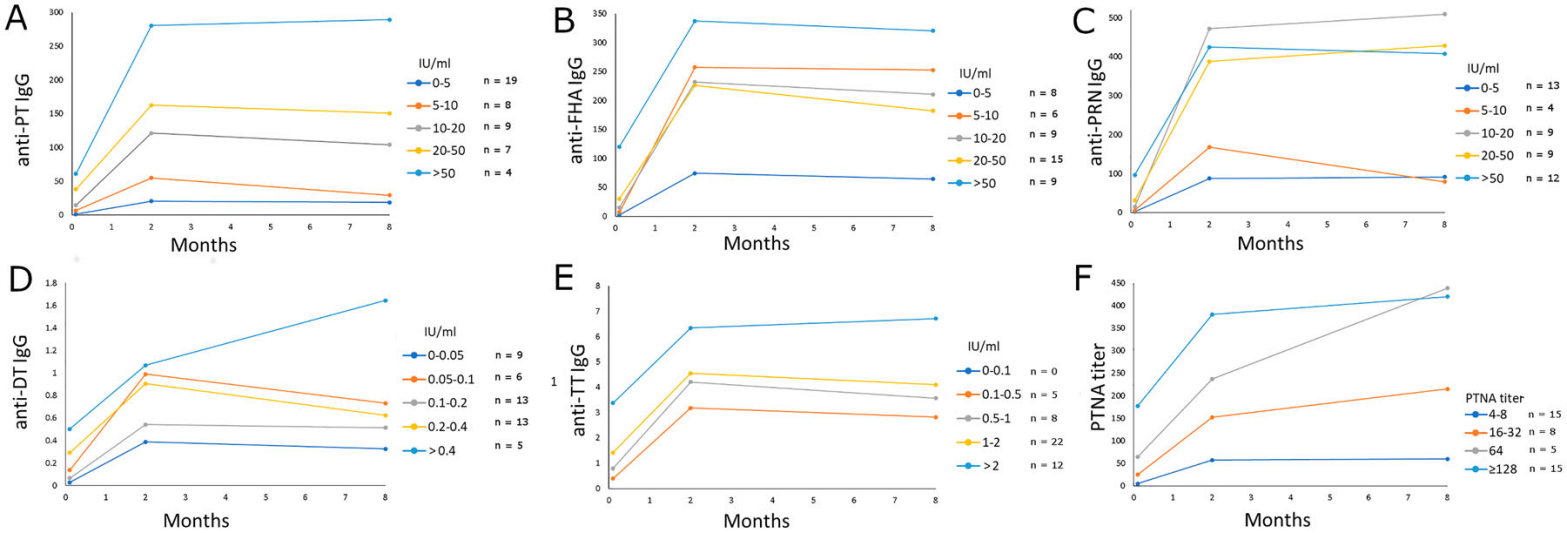
The effect of existing antibodies on the mothers’ vaccination response during pregnancy. Individuals in Arm 1 were distributed in evenly increasing increments of baseline antibody concentrations for anti-PT IgG (A), anti-FHA IgG (B), anti-PRN IgG (C), anti-DT IgG (D), anti-TT IgG (E), and PT neutralizing antibodies (F). Geometric mean concentrations of IgG antibodies and PT neutralizing antibodies are shown. Since FIM2/3 is not included in the vaccine and no anti-FIM2/3 antibody response was observed after vaccination, such analysis for FIM2/3 is not shown.

In Arm 1, IgG GMCs to all vaccine antigens were notably higher at subsequent time points if mothers had Tdap-antigen-specific antibodies before vaccination (Table 4, Figure 2). Six months after delivery, statistically significant differences were observed in PT and PRN IgG GMCs (*p* < 0.001 and *p* = 0.024, respectively) between mothers with high or low baseline antibodies. Additionally, those mothers with high anti-FIM IgG before vaccination also had significantly higher antibody concentrations to other *B. pertussis* vaccine antigens (*p* < 0.05), and thus, indicative of having a previous infection. Similarly, mothers with higher than 16 PTNA titres before vaccination had on average six-fold higher PTNAs at six months than those with lower PTNA titres (Table 4, Figure 2).

**Table 4.** Geometric mean concentrations of mothers’ IgG antibodies, pertussis toxin neutralizing antibodies (PTNA), and pertussis–specific memory B–cell frequencies before and after Tdap vaccination based on the initial baseline antibody concentrations*. Only Mothers in Arm 1 are included in the analysis.

		IgG antibodies IU/ml (95% CI)						PTNA titre	B–cell ELISPOTs per 100,000 cells (95% CI)				
Time point	* Baseline definition cutoff (pregnancy)	PT 10 IU/ml	FHA 10 IU/ml	PRN 20 IU/ml	FIM 2 IU/ml	DT 0.1 IU/ml	TT 1.0 IU/ml	(95% CI) 16 titres	PT 10 IU/ml	FHA 10 IU/ml	PRN 20 IU/ml	FIM 2 IU/ml	TT 1.0 IU/ml
**N (<= cutoff / > cutoff)**	** **	27 / 20	14 / 33	26 / 21	13 / 34	14 / 33	13 / 34	23 / 24	27 / 20	14 / 33	26 / 21	13 / 34	13 / 34
Pregnancy													
Low baseline IgG*		2.0 (1.3–3.3)	3.8 (2.3–6.2)	5.3 (3.6–7.76)	1.4 (0.8–2.2)	0.04 (0.03–0.06)	0.6 (0.5–0.8)	5.7 (4.3–7.4)	ND**	ND	ND	ND	0.4 (0.1–0.9)
High baseline IgG*	** **	27.1 (20.2–36.5)	36.7 (26.6–50.5)	59.7 (43.1–82.7)	15.8 (10.5–23.8)	0.25 (0.21–0.31)	1.9 (1.6–2.3)	96.1 (71.0–130.1)	0.7 (0.3–1.6)	0.9 (0.5–1.7)	0.4 (0.2–0.8)	ND	0.8 (0.4–1.4)
Delivery													
**Low baseline IgG**		26.8 (15.2–47.1)	126.5 (63.8–250.7)	178.3 (106.4–298.9)	1.2 (0.7–1.9)	0.57 (0.31–1.07)	3.8 (2.1–6.8)	54.4 (25.7–115.2)	0.7 (0.4–1.5)	2.9 (0.9–8.9)	2.1 (1.0–4.2)	0.3 (0.1–0.7)	1.4 (0.4–5.6)
**High baseline IgG**		158.8 (113.2–222.9)	254.6 (189.3–342.6)	408.7 (256.5–651.2)	13.9 (8.9–21.8)	0.74 (0.58–0.93)	5.1 (4.3–6.0)	244.1 (169.3–351.8)	1.4 (0.6–3.3)	4.0 (2.5–6.5)	1.9 (0.9–4.0)	ND	1.5 (0.8–2.7)
6 months													
**Low baseline IgG**		21.2 (12.7–35.3)	115.9 (61.4–218.7)	162.5 (91.7–289.9)	2.1 (1.4–3.3)	0.45 (0.25–0.81)	3.3 (1.9–5.5)	57.0 (33.9–95.8)	0.3 (0.2–0.6)	2.0 (0.6–6.3)	0.8 (0.4–1.7)	0.3 (0.1–0.7)	1.3 (0.3–4.5)
**High baseline IgG**	** **	145.3 (105.7–199.7)	221.1 (168.8–289.5)	416.7 (268.4–647.0)	21.4 (12.5–36.7)	0.66 (0.51–0.86)	4.9 (4.2–5.7)	333.0 (240.3–461.4)	1.0 (0.4–2.4)	2.0 (1.1–3.6)	1.0 (0.5–2.1)	0.3 (0.2–0.4)	1.4 (0.8–2.4)

*The definition cutoff value for low and high baseline varied for each antigen, see Figure 2.

**Not defined, GM below the lower limit of detection

For memory B cells, mothers with high anti-PT IgG before vaccination had a significantly higher number of PT-specific B-cells at six months compared to mothers with low anti-PT IgG (*p* = 0.023) (Table 4). Similarly, significantly higher numbers of memory B-cells at six months were noted for PT, FHA, and TT (*p* < 0.05) if the mothers had detectable numbers of B-cells before vaccination compared to mothers without specific memory B-cells in the circulation [18].

### Effects of pre-existing antibodies on infants’ vaccine responses

**Figure 3.**
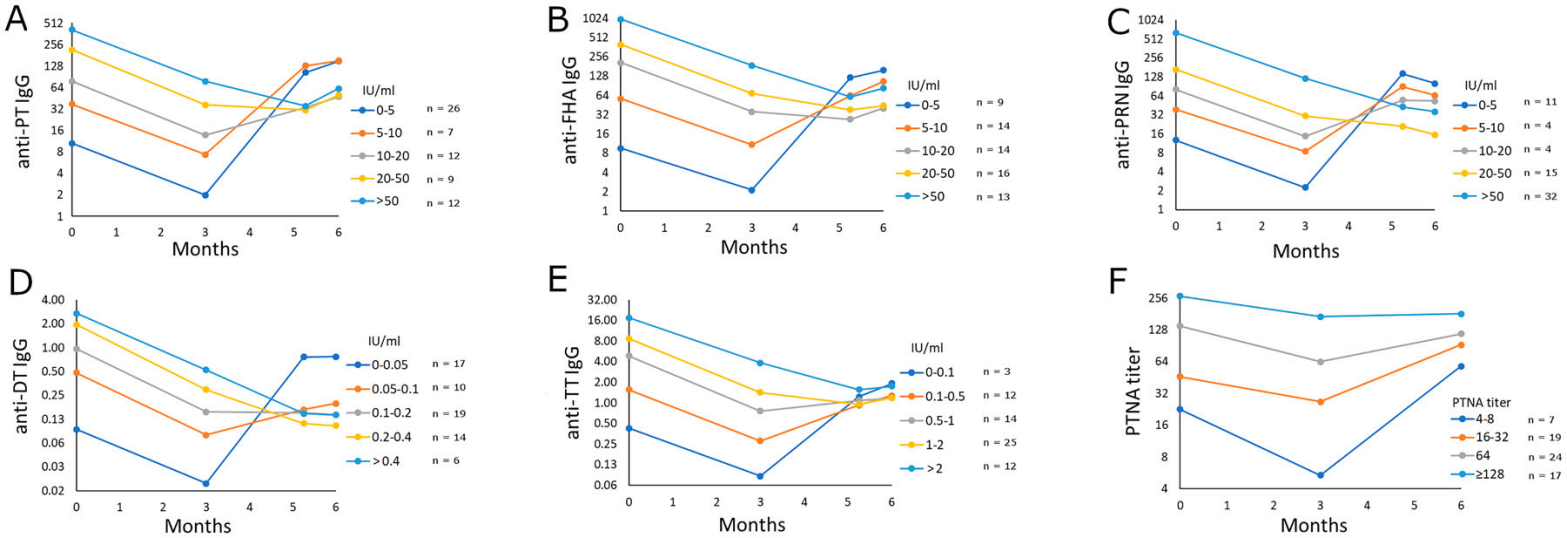
The cutoff limit for defining the possible interference caused by maternal antibodies was determined by distributing the infants in proportional increases based on antibody concentrations at three months of age. Individual cut-offs were defined for anti-PT IgG (A), anti-FHA IgG (B), anti-PRN IgG (C), anti-DT IgG (D), anti-TT IgG (E), and PT neutralizing antibodies (F). Geometric mean concentrations of IgG antibodies and PT neutralizing antibodies are shown. Since FIM2/3 is not included in the vaccine and no anti-FIM2/3 antibody response was observed after vaccination, such analysis for FIM2/3 is not shown.

The infants were further classified into groups based on their IgG GMCs at three months of age to evaluate the extent of the interference of existing antibodies. The cutoff limits for the groups of low or high baseline GMCs were determined first by distributing study subjects in proportional baseline GMCs (Figure 3). Infants with high existing antibodies had significantly fewer antibodies to all vaccine antigens, except TT at five months and one week of age after the first vaccine dose (Table 5). Although there were no statistically significant differences between the groups, notably, the first dose did not induce an increase in overall anti-TT IgG in infants with high maternal anti-TT IgG. Only after the second dose was an increase noted at six months of age. A similar trend was noted for PT and FHA antibodies. For anti-PT IgG, 100% of the infants with less than 50 anti-PT IgG IU/ml at six months of age had higher than 10 IU/ml anti-PT IgG at baseline. In contrast, infants with low baseline (maternal) antibodies had at least a twenty-fold increase in PT, FHA, PRN, and DT IgG, and a two-fold increase in TT antibodies at six months.

**Table 5.** Geometric mean values of infants’ IgG antibodies, pertussis toxin neutralizing antibodies (PTNA), and pertussis specific memory (delivery, three months, six months) and plasma (five months + seven days) B-cell frequencies before and after DTaP vaccination based on the initial baseline antibody concentrations* at three months of age.

		IgG antibodies IU/ml (95% CI)						PTNA titre	B-cell ELISPOTs per 100,000 cells (95% CI)		
Time point	*Baseline definition cutoff (3 months)	PT 10 IU/ml	FHA 10 IU/ml	PRN 20 IU/ml	FIM 2 IU/ml	DT 0.1IU/ml	TT 1.0 IU/ml	(95% CI) 16 titres	PT 10 IU/ml	FHA 10 IU/ml	PRN 20 IU/ml
**N (<= cutoff / > cutoff)**	** **	32 / 32	16 / 48	45 / 19	57 / 7	17 / 47	27 / 37	12 / 53	32 / 32	16 / 48	45 / 19
Cord blood											
Low baseline IgG*		13.5 (9.7–18.7)	17.4 (9.9–30.3)	23.2 (13.4–40.4)	6.4 (4.2–9.7)	0.09 (0.06–0.14)	4.4 (2.7–7.2)	29.9 (12.3–72.6)	ND**	ND	ND
High baseline IgG*		175.6 (130.8–235.8)	345.7 (266.9–447.8)	434.1 (329.9–571.4)	100.7 (47.2–215.1)	1.16 (0.94–1.42)	22.7 (6.8–73.7)	126.3 (90.8–175.7)	ND	ND	ND
3 months											
Low baseline IgG		2.5 (1.8–3.4)	3.6 (2.2–6.1)	4.4 (2.6–7.5)	1.3 (1.3–1.9)	0.03 (0.02–0.07)	0.6 (0.4–0.9)	8.5 (5.6–13.2)	ND	ND	ND
High baseline IgG		32.4 (24.5–42.9)	61.6 (47.9–78.2)	78.7 (61.4–100.8)	24.7 (16.0–38.1)	0.19 (0.16–0.23)	2.1 (1.6–2.8)	70.3 (58.7–84.1)	ND	ND	ND
5 months + 7d											
Low baseline IgG		107.7 (79.5–144.2)	92.2 (65.3–130.2)	100.9 (52.4–194.3)	0.4 (0.3–0.7)	0.76 (0.39–1.52)	1.2 (0.8–1.8)	–	0.9 (0.5–1.5)	1.8 (0.8–3.9)	0.8 (0.3–1.8)
High baseline IgG		33.0 (27.7–42.6)	41.6 (35.8–48.3)	34.0 (27.5–42.1)	7.8 (3.9–15.7)	0.14 (0.12–0.17)	1.7 (1.3–2.3)	–	ND	0.3 (0.2–0.4)	ND
6 months											
Low baseline IgG		148.7 (119.8–184.5)	126.7 (88.6–181.1)	87.0 (53.4–141.7)	0.3 (0.2–0.4)	0.77 (0.49–1.22)	1.3 (0.7–2.4)	68.6 (36.2–129.8)	ND	1.4 (0.6–3.1)	0.3 (0.2–0.6)
High baseline IgG		53.6 (42.8–67.2)	57.2 (45.7–71.6)	27.2 (21.7–34.0)	4.6 (2.4–8.9)	0.14 (0.11–0.18)	2.4 (1.7–3.4)	131.5 (111.3–155.3)	ND	0.4 (0.2–0.5)	ND

*The definition cutoff value for low and high baseline varied for each antigen, see Figure 3.

**Not defined, GM below the lower limit of detection

Regarding PTNAs, infants with high baseline PTNA titres (> 16) had significantly higher PTNAs at six months (*p* = 0.011) compared to infants with low baseline PTNA titres (Table 5). However, if the groups were defined instead on the basis of baseline anti-PT IgG (less or higher than 10 IU/ml), there was no difference in PTNAs between these groups (*p* = 0.826) at six months. The correlation between PTNAs and anti-PT IgG at six months was high among the infants with low baseline anti-PT IgG (Spearman R = 0.537), and non-existent in infants with high baseline anti-PT IgG (Spearman R = 0.102). In an alternative model, where the PTNA titres were divided by anti-PT IgG GMCs [16,21], it was highlighted that infants with high baseline anti-PT IgG had a significantly higher PTNA/anti-PT IgG ratio at six months (*p* < 0.001) (Supplementary Figure 1).

Frequencies of plasma B-cells were significantly higher in infants who had low baseline IgGs to PT, FHA, or PRN (Table 5) (*p* < 0.01). A marked increase was noted between baseline and six months only in infants with low baseline FHA IgG concentrations (*p* < 0.001). Proportionwise, infants with low baseline IgG GMCs had at least a frequency of five plasma B-cells for PT, FHA, and PRN in 40.0%, 41.7%, and 40.0% of cases, whereas for infants with high baseline IgG GMCs, the respective proportions were only 3.3%, 2.3%, and 15.0%. Similarly, at six months, infants with low baseline IgG GMCs had at least five memory B-cells in 3.5%, 6.7%, and 47.1% of cases, whereas for infants with high baseline IgG GMCs, the respective proportions were 3.2%, 2.2%, and 20.9%. The differences in these proportions between infants with low or high baseline IgGs were statistically significant in plasma B-cells and FHA memory B-cells (ANOVA, *p* < 0.05).

Additionally, we estimated whether antibodies in pregnant women before their vaccination (in Arm 1) could be used as a predictor for the infants’ vaccine responses. Infants born to vaccinated mothers with low baseline IgG GMCs had significantly lower IgG GMCs at three months compared to those infants born to mothers with high baseline antibody concentrations. This was observed for PT (*p* < 0.001), and PRN (*p* = 0.005). A similar trend was also noted for FHA (*p* = 0.059). Moreover, these infants had significantly higher anti-PT IgG antibodies at five and six months of age (*p* < 0.05). A similar trend was found for PT-specific plasma B-cells (*p* = 0.083) and memory B-cells (*p* = 0.028) at six months of age. However, significance was not observed for the other antigen-specific B-cells or antibodies.

## Discussion

In this study, IP resulted in high serum pre-primary DTaP vaccination antibody levels in infants. Conversely, at six months of age, antibody concentrations to PT, FHA, PRN, and DT were significantly higher in infants born to unvaccinated mothers, indicating blunting of the immune response in infants born to vaccinated mothers. In accordance with earlier data, blunting of the antibody responses, leading to lower antibody concentrations, was the strongest for PT and DT antibodies, whereas FHA and PRN responses were less affected [7,22,23]. Reassuringly, the neutralizing capacity of the PT antibodies was high in infants born to vaccinated mothers and remained at a high level after two doses of primary vaccination for up to six months.

Women with low antibody concentrations before vaccination produced lower quantities of antibodies measured at the delivery time point than women with high pre-vaccination antibody levels. To ensure relevant levels of antibody protection for the infant, more than a single booster dose could be considered for mothers with a long time since their latest vaccination and with low initial antibody levels. On the other hand, IP was unnecessary for some mothers with recent exposure history, as they had high levels of antibodies before vaccination. In comparison to a recent study with non-pregnant Finnish women of similar age (30.4 years), vaccination history, and who received the same booster, pregnant women in our study had lower antibody levels and memory B-cell frequencies to all vaccine antigens [18,24]. For this aspect, the increase of sex hormones during pregnancy has been associated with a decrease in the total concentration of pertussis antibodies [25]. Alternatively, although different sampling times between the studies influence the noted antibody concentrations due to antibody waning [20], we did not observe a significant correlation between the administration of the vaccine to the time of delivery and antibody concentrations in vaccinated mothers except for FHA antibodies (Spearman R = −0.41).

Thus, the timing of delivery may slightly hinder the study arm comparisons, particularly for FHA, as the interval between days from vaccination to delivery varied from 33 to 83 days. This may affect how much antibodies are transferred through the placenta and the antibody levels in the infants at three months of age, and consequently, further affecting infants’ vaccine responses [26]. On average, across PT, FHA, and PRN, mothers who gave birth >51 days from vaccination (n = 39) had on average roughly two-fold lower antibody levels at delivery compared to mothers who delivered the baby within 33–50 days (n = 8). However, individual variation in this analysis was high. Additionally, the mothers who had delivery within 33–50 days had higher baseline PT and FHA antibodies, which in itself was a good predictor of high antibody response. This indication of previous memory was noted in a recent vaccine booster study for neutralizing antibodies [16] as well as for B-cells [18]. Similar positive effects on the magnitude of vaccine responses in pregnant women were noted in this study. Importantly, although the effect of existing immunity was positive for the mother’s antibody responses (Figure 2), the primary antibody response of the infants was negatively associated with maternal antibody concentrations (Figure 3). The exploratory finding that mothers’ pre-vaccination immunological memory to pertussis may influence the immune status of infants both early in life due to maternal antibody transfer, as well as later in life due to blunting of antibody response to primary vaccination, warrants further study.

Indeed, infants in the control group had significantly higher antibody levels at five and six months of age to all vaccine antigens except TT, similar to what was observed by Zimmerman et al. [7]. However, a problem in the analysis of the effect of IP arises in cases of vaccinated mothers, who responded relatively poorly to the vaccine, or in unvaccinated mothers with high antibody concentrations. As an answer to this categorical problem, the data were analysed from the perspective of the infants’ antibody concentrations before the vaccine. As a result, the detected blunting of vaccination responses was further increased. Overall, the noted differences were larger between the infants categorized on the basis of low and high baseline antibody concentration (Table 5) versus that observed in the comparisons between the vaccinated and non-vaccinated mothers’ study arms (Table 3). However, TT antibody concentrations were equal between the two study groups. Presumably, as there is proportionally more TT in the vaccine compared to other antigens, the different ratios between maternal antibodies and vaccine antigens may influence the amount of blunting [27,28].

Maternally derived antibodies were also indicative of hindering infants’ early PTNA capability. Although no direct blunting was noted based on PTNA titres between the two arms, the fold increase of PTNA was significantly higher in infants born from unvaccinated mothers one month after two primary doses. Similar to our results, blunting of PTNAs was not noted earlier with DTaP vaccines [9]. However, if the comparison between infants was made based on baseline PTNA titers (>16 titers), significantly higher PTNA titres were noted at six months in those infants with high baseline PTNAs. This was explicitly noticed if the baseline was defined on the basis of PTNA titers and not anti-PT IgG concentration. Based on our analysis, those infants with low baseline antibody levels at three months of age induced relatively low neutralizing antibodies with respect to their overall anti-PT IgG levels (Table 5, Supplementary Figure 1). Conversely, vaccinated mothers had a remarkably high PTNA–to–anti-PT IgG ratio of nearly 4. As a result, it can be deduced that most of the noted high neutralizing capability in infants was related to maternal antibodies and not to those induced by infants’ primary vaccination. Altogether, this novel finding further emphasizes the importance of personalized characterization of study subjects from many viewpoints to follow up on the effects of existing antibodies, both from the side of the mothers and infants, instead of solely relying on IgG measurements. Although the negative association between existing antibodies and infants’ vaccination responses has been reported long ago [28,29], and it has been acknowledged in the study design, the direct application of this knowledge in analysis methods should be further emphasized in research.

Another clear indication of maternal antibody blunting was noted in the early B-cell development of the infants to pertussis antigens, of which there exist no previous studies to the authors’ knowledge. In mice, high maternal antibodies to haemagglutinin of Influenza A virus or TT were demonstrated to limit the expansion of Tfh cells and the differentiation of germinal centre B-cells into memory or plasma cells [30]. The acellular vaccination in our study had in general relatively weak success in inducing plasma and particularly memory B-cells even without the interference of maternal antibodies, both in pregnant women and infants. The overall prevalence of antigen-specific plasma B-cells and FHA-specific memory B-cells was markedly reduced in infants with existing maternal antibodies. Of the vaccine antigens studied, FHA was the most immunogenic antigen to induce memory B-cells, hypothetically due to its large size and high quantity in the vaccine.

Regarding study limitations, the number of study subjects in this study was limited due to COVID pandemic restrictions. Additionally, a few individual time points were missed due to challenging sampling from the infants and restricted blood volumes, leading to prioritization among assays. Consequently, although we recognized clear indications of the effect of existing immunity and trends of blunting, many observations were left without statistical significance. The infants were followed in this study for up to six months, and the long-term impact of these findings remains to be confirmed. Thus, we encourage the use of the presented analytical approaches to already existing larger cohort data and for future studies.

In conclusion, after IP, maternal antibodies were found in high quantities for up to three months post-delivery and provided antibody-mediated protection for infants. The maternal response to the vaccine was enhanced if the mothers had indications of existing memory, in this case antibodies or detectable memory B-cells, to the vaccine antigens. However, the maternal antibodies interfered with the quantity of antibodies and plasma B-cells induced by the vaccination in infants. The highest fold increase of PTNAs was also noticed in infants born from unvaccinated mothers in comparison to infants born from vaccinated mothers after vaccination. Moreover, the noted effect of blunting was the strongest in infants with high maternal antibodies. In this study, exploratory cutoff levels were characterized and defined, resulting in the antibody blunting of infants. Importantly, comparisons between the infants using baseline antibody definitions instead of the study arms established a more in-depth analysis of the effect of maternal antibodies. These results show that an integrated analysis of both individual and group responses is needed in future pertussis vaccine studies. As a result, our findings may help to explain possible differences between studies and determine what kind of impact vaccine failures or previous infections pose to the interpretation of such studies. Based on existing antibody concentrations before vaccination, it may be possible to identify mothers who do not need a single dose, and likewise those mothers who would need an additional booster dose. Both aspects are important to ensure sufficient protection for infants and to avoid unnecessarily high levels of antibodies, which blunt the primary vaccination response of infants.
